# The spread of SARS-CoV-2 at school through the different pandemic waves: a population-based study in Italy

**DOI:** 10.1007/s00431-022-04654-x

**Published:** 2022-10-21

**Authors:** Ilaria Pistellato, Marco Fonzo, Andrea Calzavara, Paola Sorrentino, Vittorio Selle, Luca Gino Sbrogiò, Chiara Bertoncello

**Affiliations:** 1 Department of Prevention, Local Health Authority 3, 30174 Venice, Italy; 2 Hygiene and Public Health Unit, Department of Cardiac Thoracic and Vascular Sciences and Public Health, University of Padova, 35131 Padova, Italy

**Keywords:** SARS-CoV-2, COVID-19, School, Educational setting, Screening, Distance learning

## Abstract

Proactive school closures are often considered an effective strategy by policy-makers and the public to limit SARS-CoV-2 transmission. While evidence on the role of students in the spread is debated, the effects of closures on children's well-being are well known. In the light of this, we aimed to assess viral spread in educational settings, by calculating the rate of secondary infections per school class and identifying factors associated with cluster generation. We conducted a combined longitudinal and cross-sectional population-based study between October 2020 and November 2021. Secondary screening was conducted whenever a SARS-CoV-2 positive subject had been in the school environment in 48 h prior to symptoms onset or on the date of swab, if asymptomatic. The effect of selected variables on COVID-19 cluster generation was assessed by logistic regression. We identified 1623 primary COVID-19 cases. Of these, 72.5% resulted in no secondary case, 15.6% in 1, and 11.9% in 2 + . The probability of generating a 2 + cluster was lower when the index case was a student, rather than school staff (AOR = 0.42; 95%CI: 0.29–0.60). The number of clusters per week was in line with COVID-19 incidence trend in the general population.

*Conclusions*: Index cases at school led to no secondary case in about three out of four times and only to a secondary case in about 15%. School environment does not facilitate viral spread, but rather reflects circulation in the community. Appropriate measures and timely monitoring of cases make school a safe place. Given the effects on children’s learning and well-being, it is essential to favour school attendance over distance learning.

**Table:** 

**What is Known:** • *During the COVID-19 pandemic, most European countries resorted to school closures to counter viral transmission.* • *Although the scientific debate on the suitability of school closures as a non-pharmaceutical intervention is still open and the role of school children in facilitating the spread of SARS-CoV-2 is not supported by unequivocal evidence, there is now a growing awareness of the impact on children's well-being.*	
**What is New:** • *The contribution of educational settings and students in facilitating viral spread appears limited, as exposure to a positive individual in the school environment led to no secondary cases among students in 72% of cases and only one secondary case in about 15%.* • *The likelihood of generating school clusters was approximately halved when the index case was a student compared to teachers or other school personnel.*

**What is Known:**

• *During the COVID-19 pandemic, most European countries resorted to school closures to counter viral transmission.*

• *Although the scientific debate on the suitability of school closures as a non-pharmaceutical intervention is still open and the role of school children in facilitating the spread of SARS-CoV-2 is not supported by unequivocal evidence, there is now a growing awareness of the impact on children's well-being.*

**What is New:**

• *The contribution of educational settings and students in facilitating viral spread appears limited, as exposure to a positive individual in the school environment led to no secondary cases among students in 72% of cases and only one secondary case in about 15%.*

• *The likelihood of generating school clusters was approximately halved when the index case was a student compared to teachers or other school personnel.*

## Introduction

Policy-makers and the public often believe that proactive closure of school environments is one of the effective strategies to limit the spread of SARS-CoV-2 within the community [1]. Reasonably, studies exploring educational settings and households are crucial in understanding the role of students in viral transmission [2, 3]. However, the scientific debate on the use of school closures as a non-pharmacological intervention is still open, and to date, there is growing awareness of the impact on children’s well-being.

Some researchers have concluded that the risk of contracting COVID-19 is higher in adults living together with children attending school [4, 5]. On the other hand, a large cohort study conducted in the UK showed that the risk of SARS-CoV-2 infection in adults living in the same household with children aged 11 or younger was in line with the rest of the population [6], although the risk of both infecting and being infected was slightly higher for adults living with children aged 12–18 years. However, this increase was not matched by a higher lethality rate, and no significant association was found between school closures and the course of the epidemic. Similarly, a case–control study conducted in Italy showed that the risk of infection in children was higher for those who had family rather than school contact [7]. As shown in a preprint systematic review, viral spread at school is minimal when appropriate preventive measures are implemented [8]. Overall, available epidemiological data suggest that the reopening of schools does not appear to be associated with a substantial increase in the incidence in the general population, when mitigation measures in the education settings are correctly implemented [9].

During the first COVID-19 wave, most European countries resorted to school closure. In Italy, the closure of all school grades was imposed on 4 March 2020 by governmental decree (DPCM 23/2/2020). Schools remained closed until the end of the school year (i.e., early June) and reopened in September 2020, when a national protocol was enforced. Recommendations included body temperature measurement and hand hygiene at the school entrance; unidirectional flows of students; compulsory face mask in common areas for all school staff members and students and for teachers and high school students also when seated (from November 6 2020, this mask mandate was extended to students also when seated); 1-m compulsory distance between seats; frequent natural ventilation; and ban or reduction of sports and music activities and reduced duration of lessons [10]. However, due to the increase in the number of contagions at the general population level, in-person school attendance alternated with periods of distance learning throughout the whole 2020–2021 school year, with slight differences depending on the school grade.

The debate on school closure and the consequent resort to distance learning continues to be a debated topic, especially if considering that the number of secondary cases identified per class is currently one of the main criteria for imposing distance learning. Since the beginning of the pandemic, this threshold has changed over time. As of February 2022, classes in primary schools remained in attendance until there were less than four secondary cases per class; in middle and high schools, activities remained in attendance until there was a single secondary case (DL 4/2/2022).

 The present study aimed to calculate the rate of secondary infections per school class between October 2020 and November 2021, considering the strategic importance of this indicator in public health decision-making. Secondly, the study aimed at identifying factors potentially associated with the development of school clusters. Finally, the time course of school clusters was compared with the incidence of COVID-19 infection in the general population by age in the same area.

## Methods

We conducted a combined longitudinal and cross-sectional population-based study between October 2020 and November 2021. We included in the study the schools in the province of Venice, a catchment area of approximately 618,000 inhabitants (of which 70,800 in the 6–18 age group), under the control of the Local Health Unit “AULSS 3 Serenissima”. In the considered period, primary and middle schools remained open for almost the entire time, with the exception of a 3-week stop (15 March 2021 to 6 April 2021). High school students were obliged to distance learning from 6 November 2020 to 1 February 2021 and then allowed to partially attend school (in a proportion varying from 50 to 75% of activities) until 26 April. Subsequently, the attendance proportion gradually increased from 75 to 100% until the end of the school year. It is noted here that school attendance was also guaranteed during the aforementioned 3-week stoppage in the case of workshops or students with disabilities and special educational needs, with the mandatory use of respiratory protective equipment. In September 2021, schools at all levels reopened without distance learning.

In accordance with national guidelines, school staff or students who acquired COVID-19 outside the school environment had to promptly inform the school management, which had to coordinate with the Local Health Unit to carry out secondary screening among staff and students (or impose a 14-day quarantine with a swab before reintegration into school).

Although regulations issued at national level allowed for either secondary screening or quarantine, we systematically performed secondary screening by a first swab at T_0_ and a second swab at T_10_ (10 days later), except for the period between 10 January and 11 February, which was excluded from the study. Secondary screening at school was conducted whenever a positive subject had been in the school environment within 48 h prior to the onset of symptoms (or the date of the swab, if asymptomatic). All students and staff members present in the same classroom (or engaged in the same activities) were screened for SARS-CoV-2. In our study setting, rapid antigen tests were used during 2020, while on the occasion of the third wave, they were replaced by third-generation rapid antigen tests. In fact, the Regional Health Authority allowed for either a third-generation rapid antigen test or a molecular swab (Nota Reg. Veneto n. 108,713 8/3/2021). Information was collected on the school grade, the index case (student or school staff), and vaccination status of the index case. All data on positivity reports in the school setting were collected in an ad hoc database and implemented on a daily basis. Population incidence data for SARS-CoV-2 positivity were retrieved from the local database.

The size of identified school clusters was classified as follows: “no secondary cases”, “only 1”, and “2 or more”. Differences in the proportion of cluster size by time, school grade, type, and vaccination status of the index case were evaluated by mean of Fisher’s exact tests; *p* values were also reported. We defined as “vaccinated” those who received the second dose (or single dose for the *Jcovden* COVID-19 vaccine by Janssen) at least 2 weeks earlier. The potential effect of the aforementioned variables on the generation of a cluster of 1 or 2 + secondary cases was assessed with multinomial logistic regression adjusting for time (either 2nd, 3rd, or 4th pandemic wave), school grade, type, and vaccine status of the index case; adjusted odds ratios (AOR) and relative 95% confidence intervals were calculated. Statistical analyses were performed with IBM SPSS version 27. The incidence of SARS-CoV-2 infection in the general population residing in the investigated area was plotted together with the incidence in the age groups of interest (i.e. 6–10, 11–13, and 14–19 years). The number of notifications received per type of school and the number of 2 + clusters were also reported.

This study complies with the Declaration of Helsinki. Data used in the present study were collected as part of the local epidemic surveillance by the Local Health Authority, and the need for patients’ consent was waived due to the anonymous nature of data. Data shown in this study were extracted, anonymised, and handled in this form for all the purposes of the present research. According to the Italian legislation (DM 18/3/1998), data can be analysed and used in aggregate form for scientific purposes without further authorization, meaning that no formal ethical committee approval was needed.

## Results

Between October 2020 and November 2021, 1623 COVID-19 notifications were collected. Of these, 1176 (72.5%) did not lead to any secondary case in the school setting, as shown in Table 1. More in detail, there were 253 (15.6%) clusters with 1 secondary case, 94 (5.8%) with 2, 63 (3.9%) with 3 or 4, 19 (1.2%) with 5 or 6, and 18 (1.1%) with 7 or more secondary cases.

**Table 1:** Distribution of SARS-CoV-2 secondary cases per school class. Italy, 2020–2021

		Total		Secondary cases per school class						
				0		1		2 +		
		$n$	%	$n$	%	$n$	%	$n$	%	$p$^*^
Total		1623	100,0%	1176	72.5%	253	15,6%	194	12.0%	
Pandemic wave	Oct–Dec 2020 (2nd wave)	844	52.0%	648	76.8%	121	14,3%	75	8.9%	0.000
	Feb–June 2021 (3rd wave)	434	26.7%	290	66.8%	78	18,0%	66	15.2%	
	Sept–Nov 2021 (4th wave)	345	21.3%	238	69.0%	54	15,7%	53	15.4%	
School grade	Primary	646	39.8%	458	70.9%	96	14,9%	92	14.2%	0.010
	Middle	519	32.0%	362	69.7%	93	17,9%	64	12.3%	
	High	458	28.2%	356	77.7%	64	14,0%	38	8.3%	
Index case	Student	1289	79.4%	952	73.9%	202	15,7%	135	10.5%	0.001
	Teacher or other school staff	334	20.6%	224	67.1%	51	15,3%	59	17.7%	
Index case vaccine status	Non vaccinated	1579	97.3%	1143	72.4%	246	15,6%	190	12.0%	0.938
	Vaccinated	44	2.7%	33	75.0%	7	15,9%	4	9.1%	

^*^Fisher's exact test

In the so-called second wave (October–December 2020), the proportion of 2 + clusters was 8.9%, lower than in the third (February–June 2021) and the fourth wave (September–November 2021) when it increased to 15.2% and 15.4%, respectively (*p* < 0.001). While 70.9% and 69.7% of notifications were not followed by any secondary infection in primary and middle schools, respectively, in high school, this percentage was 77.7% (*p* = 0.010). When the index case was a teacher (or other school staff), the development of a 2 + cluster occurred in 17.7%, whereas it occurred in a significantly lower percentage (10.5%, *p* = 0.001) when the index case was a student. Only 44 (3%) index cases were vaccinated against SARS-CoV-2; of these, 21 were school staff members and 23 students, 17 of whom were of high school age. Within the local surveillance system, there were no missing data for any of the investigated variables.

As shown in Table 2, the results of the logistic regression demonstrate that the second wave was associated with a lower occurrence of 2 + clusters (AOR = 0.37; 95%CI: 0.24–0.56) compared to the fourth wave, while no significant difference was noted compared to the third wave. Both primary (AOR = 1.74; 95%CI: 1.16–2.63) and middle schools (AOR = 1.76 95%CI: 1.14–2.72) showed a higher risk than high schools for the involvement of two or more secondary cases. The involvement of 2 + secondary cases was also less associated with the index case being a student rather than a school staff member (AOR = 0.42; 95%CI: 0.29–0.60).

**Table 2:** Multinomial logistic regression. Risk of school cluster generation after SARS-CoV-2 diagnosis. Adjusted odds ratios, 95% confidence intervals. Italy, 2020–2021

		Ref. No secondary cases					
		Only 1 secondary case			2 + secondary cases		
		AOR	95% CI		AOR	95% CI	
Pandemic wave	Oct–Dec 2020 (2nd wave)	0.78	0.53	1.15	0.37	0.24	0.56
	Feb–June 2021 (3rd wave)	1.19	0.79	1.78	0.86	0.56	1.31
	Sept–Nov 2021 (4th wave)	ref			ref		
School grade	Primary	1.17	0.82	1.66	1.74	1.16	2.63
	Middle	1.48	1.04	2.11	1.76	1.14	2.72
	High	ref			ref		
Index case	Student	0.85	0.60	1.22	0.42	0.29	0.60
	Teacher or other school staff	ref			ref		
Index case vaccine status	Non vaccinated	1.06	0.43	2.60	2.89	0.94	8.90
	Vaccinated	ref			ref		

**Fig. 1:**
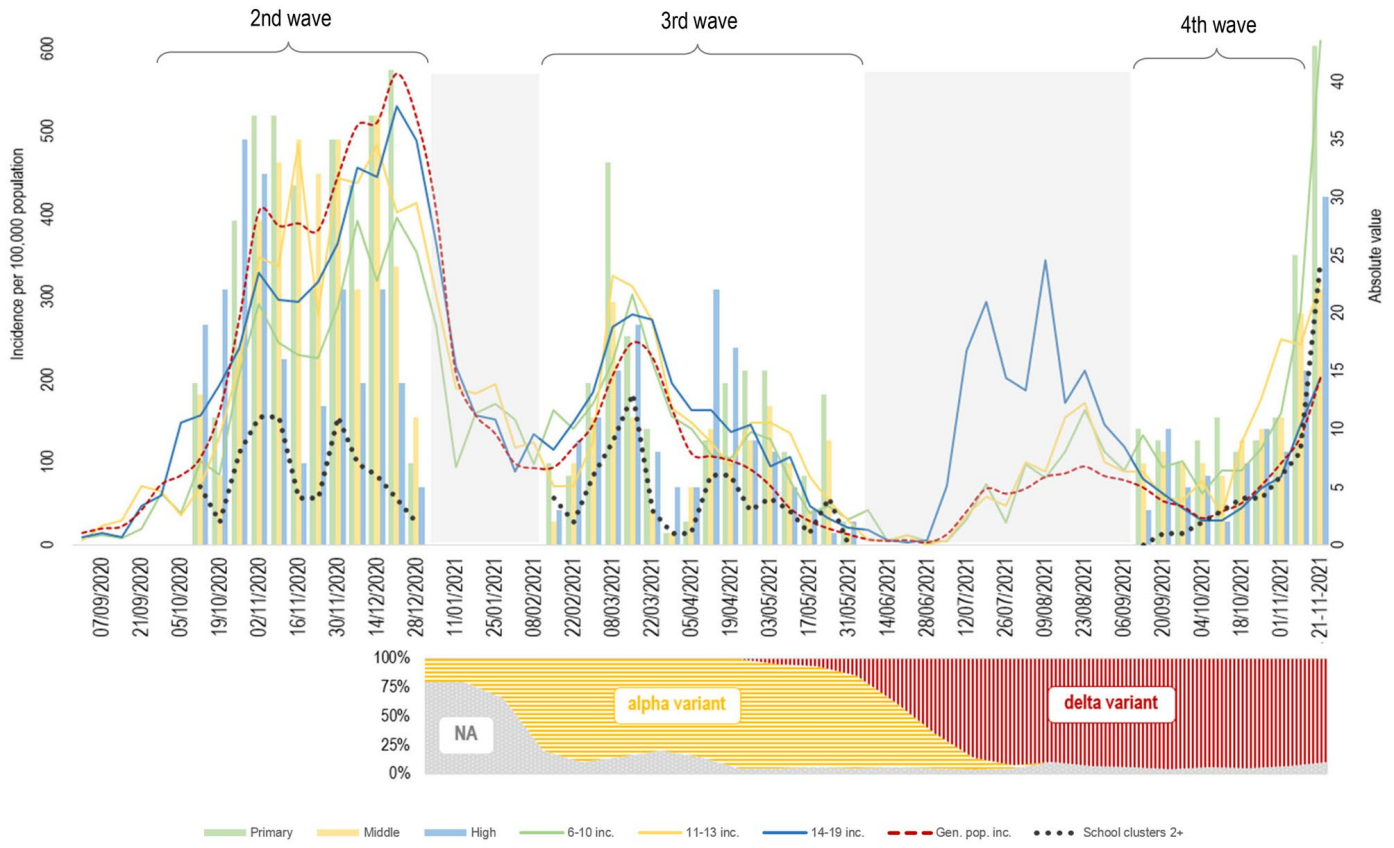
Time trend of notifications at school of SARS-CoV-2 positivity (bars); general population incidence and incidence in the age groups 6–10, 11–13, and 14–19 (lines); number of school clusters with 2 + secondary cases (thick dotted line). The grey areas represent periods either not included in this study or on school vacation. Graph below: prevalence of main variants by time in Italy (modified from Italian National Institute of Health, report n. 16 of 19 January 2022). Italy, 2020–2021

The number of notifications per week during the second wave was higher than during the third, as shown in Fig. 1. Similarly, the population incidence in the three age groups examined was higher in the second wave than in the third. When schools reopened in mid-September, the incidence in both the school-age population and general population was lower than during the summer, while at the end of the observation period, a sharp increase in incidence was noted in the general population and an even sharper increase in the 6–10 and 11–13 age group. The number of 2 + clusters per week followed a time course in line with the incidence in the general population, with a two-peak trend in both the second and third waves and a sharp increase between September and November 2021.

## Discussion

Trying to quantify the contribution of each of the tools implemented at both the population and school level in limiting the spread of SARS-CoV-2 is certainly a difficult task. This issue becomes even more difficult when considering the role of additional factors, such as a potential difference in susceptibility or transmissibility among the school-age population or variations in social interactions over time outside the school environment. However, our results show that the school environment as a whole does not seem to facilitate viral spread: The identification of a potential index case through contact tracing was not followed by the generation of any secondary case in about three out of four times or limited to a single secondary case in about 15% of the times.

The most recent evidence shows that children and adults have a comparable susceptibility to SARS-CoV-2, although severe presentations are less common among younger children. However, it seems that nursery and primary school children transmit the virus less frequently than adolescents and adults. To date, seroprevalence data support that there are no significant differences between the different age groups and in the school population [11]. The advent of new viral variants has led to an increase in transmissibility in all age groups; at the same time, the advancement of the vaccination campaign in the adult and elderly population has led to a decrease in susceptibility in these age groups, resulting in a relatively higher prevalence among children [12]. Several studies analysed the transmission capacity of the virus in children compared to adults, examining the dynamics of intrafamilial transmission. These studies found that children and adolescents were rarely identified as index cases [13, 14]. Furthermore, the rate of secondary attacks correlated with age, being lowest in children up to 11 years, slightly higher between 12 and 17 years and highest above 18 years [15, 16]. Other authors found that the opening of schools had no impact on mortality or severity of COVID-19 and that children were rarely index cases, when infection occurred in cohabitants [6, 17].

The increased number of school outbreaks at epidemic peaks in the general population raises the possibility that secondary cases are not actually linked to the school’s index case. Transmission in schools does not seem to be the determinant factor for transmission in the community [10, 18–20]. On the contrary, the spread of the virus within schools seems to be quite limited [21–24]. The results of our study suggest that schools do not amplify the transmission of SARS-CoV-2, but rather reflect the level of transmission in the community.

Current evidence shows that secondary infections at school are more frequent when the index case is a school staff member rather than a student [10] and school outbreaks are more frequent in regions where the incidence of the disease is high [25]. Our study confirms this result and lays the foundations for a proposal for regular screening of teaching and non-teaching staff, the efficiency of which could be proportionally greater than a screening programme of the entire school population.

Our data show that in the so-called second wave (October–December 2020), the probability of generating a 2 + cluster was lower than in the last wave, despite a higher incidence in the school-age population. This apparent paradox may be due to several reasons. Firstly, on the occasion of the third wave onwards, third-generation rapid antigen tests with higher sensitivity were used. Secondly, in the last wave we analysed, the delta variant was prevalent nationwide, and its higher transmissibility may have played a role in determining this result. Thirdly, it should be mentioned that during the last considered period, no particular restrictions were imposed on children for sports, music, or other activities external to the school setting, increasing the possibility that secondary cases at school may have had an extracurricular origin. Fourthly, as also highlighted by the ECDC, reported cases in children also depend on the frequency of testing in this age group, which has varied over time and between countries. It is likely that testing approaches in schools have been expanded over time with the wider implementation of rapid antigen detection tests and in an effort to keep schools open, and a similar dynamic likely took place in the investigated context [9].

The COVID-19 epidemic had a negative impact not only on the physical and psychological health and learning of children and adolescents, but also on school drop-out, affecting especially disadvantaged families and contributing to worsening health and educational inequalities [26]. A pre-print systematic review including 72 studies from 20 countries shows a lowering of the stress tolerance threshold and an increase in post-traumatic symptoms (anxiety and depression) and suicidal ideation in the 13–18 age group [27]. Not surprisingly, the American Academy of Paediatrics, the American Academy of Child and Adolescent Psychiatry, and the Children's Hospital Association declared a national state of emergency for children's mental health in 2021[28]. Enzgell estimates a worsening of school performance by 20% after only 2 months of school closure; this effect occurs especially in less educated families and in students belonging to fragile categories [29]. In Italy, the national survey on student learning performance (INVALSI) showed a worsening of learning in Italian and mathematics in third and fifth grade students compared to 2019 [30]. This deterioration occurred particularly in students with families in disadvantaged economic situations. In English, there was no significant loss of learning compared to 2019, but there was a strong difference between the 5 different regional macro-areas in the achievement of the minimum level set. Growing data on early school leaving in Italy, although partial, emerge from the reports collected by the juvenile prosecutors' offices, especially in Southern Italy.

Our study presents some limitations. The three epidemic waves are not perfectly comparable due to the regulatory changes that took place and the prevalence of the different viral variants over time, but secondary screening was conducted uniformly over the periods considered, through the application of a shared protocol. Our secondary screening activity may overestimate the transmission in the school setting because not all secondary cases may be attributable to the index case. We were unable to discriminate whether secondary positivities revealed by screening swabs were really the results of infections occurring in the school setting or whether they were due to infections occurring in social contexts outside school. However, although this may have overestimated the phenomenon, the transmission of SARS-CoV-2 in the school environment remained limited. Our study ended before the introduction of the vaccine in the age group 5–11, and those aged between 12 and 19 only had the opportunity to receive the vaccine in the last period considered in our study (the coverage achieved at the end of the study period was 67%). In light of this, we can say that we considered somehow an even worse scenario and there is a substantial possibility that our study, again, did not underestimate the phenomenon. A noteworthy strength is that, although nationally enacted regulations required one between secondary screening and quarantine, in our context, secondary screening was systematically performed with a first swab at T_0_ and a second swab at T_10_.

In conclusion, our results support the evidence outlining school as a safe place, certainly without neglecting the fundamental contribution of adopting appropriate measures (use of face masks, interpersonal distancing, frequent hand and respiratory hygiene), together with timely monitoring of cases. Given the documented negative effects of school closures not only on learning, but also on students’ physical, emotional, and relational spheres, it is essential to maintain school attendance and consider school closures as a last resort, reactive rather than proactive.
